# Low-dose corticosteroid therapy for cardiogenic shock in adults (COCCA): study protocol for a randomized controlled trial

**DOI:** 10.1186/s13063-021-05947-6

**Published:** 2022-01-03

**Authors:** Armand Mekontso Dessap, François Bagate, Clément Delmas, Tristan Morichau-Beauchant, Bernard Cholley, Alain Cariou, Benoit Lattuca, Mouhamed Moussa, Nicolas Mongardon, Damien Fard, Matthieu Schmidt, Adrien Bouglé, Mathieu Kerneis, Emmanuel Vivier, François Roubille, Matthieu Duprey, Véronique Decalf, Thibaud Genet, Messaouda Merzoug, Etienne Audureau, Pierre Squara

**Affiliations:** 1 Assistance Publique-Hôpitaux de Paris (AP-HP), Hôpitaux Universitaires Henri-Mondor, Service de Médecine Intensive Réanimation, F-94010 Créteil, France; 2 Univ Paris Est Créteil, CARMAS, F-94010 Créteil, France; 3 Univ Paris Est Créteil, INSERM, IMRB, F-94010 Créteil, France; 4 Intensive Cardiac Care Unit, Rangueil University Hospital, Toulouse, France; 5 Centre Cardiologique du Nord, service de réanimation, St Denis, France; 6 AP-HP, Hôpital Européen Georges Pompidou, Service d’Anesthésie-Réanimation, Paris, France; 7 AP-HP, Centre-Université de Paris, Hôpital Cochin, Service de Médecine Intensive Réanimation, Paris, France; 8 Hôpital Universitaire de Nîmes, Unité de soins Intensifs cardiologique, Nîmes, France; 9 CHU de Lille, Service d’Anesthésie-Réanimation cardiovasculaire, Lille, France; 10 U955-IMRB, Equipe 03 “Pharmacologie et Technologies pour les Maladies Cardiovasculaires (PROTECT)”, Inserm, Univ Paris Est Créteil (UPEC), Ecole Nationale Vétérinaire d’Alfort (EnVA), F-94700 Maisons-Alfort, France; 11 AP-HP, Hôpitaux Universitaires Henri Mondor, Service d’anesthésie-réanimation chirurgicale, DMU CARE, DHU A-TVB, F-94010 Créteil, France; 12 AP-HP, Hôpitaux Universitaires Henri-Mondor, Unité de soins Intensifs cardiologique, F-94010 Créteil, France; 13 AP-HP, Hôpital Pitié-Salpétrière, Service de Médecine Intensive Réanimation, Paris, France; 14 AP-HP, Hôpital Pitié-Salpétrière, Service d’Anesthésie-Réanimation cardiovasculaire, Paris, France; 15 AP-HP, Hôpital Pitié-Salpétrière, Unité de soins Intensifs cardiologique, Paris, France; 16 Centre Hospitalier Saint Joseph Saint Luc, Service de Médecine Intensive Réanimation, Lyon, France; 17 PhyMedExp, Université de Montpellier, INSERM, CNRS, Cardiology Department, CHU de Montpellier, INI-CRT, Montpellier, France; 18 Grand Hôpital de l’Est Francilien, Hôpital Jossigny, Service de Médecine Intensive Réanimation, Marne la Vallée, France; 19 Centre Hospitalier de Pontoise, Unité de soins Intensifs cardiologique, Pontoise, France; 20 CHU de Tours, Unité de soins Intensifs cardiologique, Tours, France; 21 CMC Ambroise Paré, département de recherche clinique, Neuilly-sur-Seine, France; 22 AP-HP, Hôpitaux Universitaires Henri-Mondor, Service de recherche clinique, F-94010 Créteil, France; 23 CMC Ambroise Paré, service de réanimation, Neuilly-sur-Seine, France

**Keywords:** Corticosteroid, Hydrocortisone, Fludrocortisone, Adrenal insufficiency, Cardiogenic shock

## Abstract

**Background:**

Cardiogenic shock (CS) is a life-threatening condition characterized by circulatory insufficiency caused by an acute dysfunction of the heart pump. The pathophysiological approach to CS has recently been enriched by the tissue consequences of low flow, including inflammation, endothelial dysfunction, and alteration of the hypothalamic-pituitary-adrenal axis. The aim of the present trial is to evaluate the impact of early low-dose corticosteroid therapy on shock reversal in adults with CS.

**Method/design:**

This is a multicentered randomized, double-blind, placebo-controlled trial with two parallel arms in adult patients with CS recruited from medical, cardiac, and polyvalent intensive care units (ICU) in France. Patients will be randomly allocated into the treatment or control group (1:1 ratio), and we will recruit 380 patients (190 per group). For the treatment group, hydrocortisone (50 mg intravenous bolus every 6 h) and fludrocortisone (50 μg once a day enterally) will be administered for 7 days or until discharge from the ICU. The primary endpoint is catecholamine-free days at day 7. Secondary endpoints include morbidity and all-cause mortality at 28 and 90 days post-randomization. Pre-defined subgroups analyses are planned, including: postcardiotomy, myocardial infarction, etomidate use, vasopressor use, and adrenal profiles according the short corticotropin stimulation test. Each patient will be followed for 90 days. All analyses will be conducted on an intention-to-treat basis.

**Discussion:**

This trial will provide valuable evidence about the effectiveness of low dose of corticosteroid therapy for CS. If effective, this therapy might improve outcome and become a therapeutic adjunct for patients with CS.

**Trial registration:**

ClinicalTrials.gov, NCT03773822. Registered on 12 December 2018

## Background

Cardiogenic shock (CS) is a serious condition characterized by circulatory insufficiency caused by an acute dysfunction of the heart pump. The prognosis of critically ill patients with CS has relatively improved over these last decades [1], thanks to recent progress [2], like early myocardial revascularization during myocardial infarction [3]. Nevertheless, the early mortality rate remains unacceptably high, around 45% [1, 2].

The pathophysiology of CS has recently evolved with a new hemodynamic paradigm [4] involving sepsis-like alterations, with the tissue consequences of low flow, including inflammation, endothelial dysfunction, and alteration of the hypothalamic-pituitary-adrenal (HPA) axis. Critical illness-related corticosteroid insufficiency (CIRCI) was first described in septic shock [5] and is characterized by a functional impairment of the HPA axis during various critical illnesses [6]. Corticosteroids have pleiotropic effects including immune modulation, metabolic, and cardiovascular effects. The results of studies testing corticosteroid supplementation in septic shock were controversial in terms of mortality [7–10], but most of them demonstrated that corticosteroid therapy accelerates reversal of shock. Two large recent trials (ADRENAL [11] and APROCCHSS [12]) confirmed a faster reversibility of septic shock with low-dose steroids. The latest study further demonstrated a decrease in mortality in the group receiving a combination of fludrocortisone and hydrocortisone in adult patients with severe septic shock.

During septic shock, CIRCI seems to be more prevalent in patients with myocardial dysfunction as compared to those with normal cardiac function [13]. CIRCI has recently been found in patients with CS and associated with a worse prognosis in this setting [14, 15]. However, no study has evaluated the effect of corticosteroid supplementation in CS.

Although the survival could be a more robust primary endpoint, it seemed unreasonable for the present study given the small difference in mortality expected based on septic shock studies. A composite endpoint including mortality and shock reversal seemed more appropriate.

## Objective

The “low dose COrticosteroids for Cardiogenic shoCk in Adult patients” (COCCA) trial is designed to evaluate the hemodynamic effects of early low-dose corticosteroid therapy (with hydrocortisone and fludrocortisone) on CS reversal, as defined by catecholamine-free days at day-7.

## Method

### Study design

The COCCA trial is a nationwide multicenter, randomized, double-blind, placebo-controlled trial, with 2 parallel arms.

### Ethical consideration and clinical trial authorization

This study follows the principles of the current version of the Helsinki Declaration, the French Law on Protection of Personal Information and the National Health Law. The whole protocol has been reviewed and approved by an Independent Ethics committee (Comité de Protection des Personnes CPP— Ile-de-France IV, number 2018/40) and the French health authorities (Agence Nationale de Sécurité du Médicament, on September 3, 2018, number EudraCT 2018-000729-30). The trial was registered at ClinicalTrials.gov on December 12, 2018, before inclusion of the first patient, under the number NCT03773822.

### Participating centers

Participating centers include French university and non-university hospitals, all with a cardiac and a medical intensive care unit (ICU) and all with a high volume of patients with CS. List of the participating centers is provided in Additional file 1.

### Participants

Eligible patients are all consecutive adult patients who are admitted to the participating ICUs for CS. Complete eligible criteria are shown in Table 1. All eligible patients will be included in the study after obtaining signed informed consent or adapted procedure as per French law. In practice, the consent will be obtained as follows:

**Table 1:** Eligibility criteria

Inclusion criteria	Exclusion criteria
1. Adult (age **≥** 18 years);	1. Cardiogenic shock state with catecholamine infusion for more than 24 h;
2. Cardiogenic shock state according to the following definition:	2. Presence of sepsis at inclusion;
a. Systemic arterial hypotension (SAP < 90 mmHg or MAP < 65 mmHg for 30 min) and/or low cardiac output requiring catecholamines to achieve a systolic blood pressure ≥ 90 mmHg;	3. Cardiac arrest recovered within 7 days prior to inclusion, with at least one of the following early signs of poor prognosis: no witness, non-shockable rhythm, CAHP score > 150 [16];
a. Sign of hypocontractility or low cardiac output among the following: cardiac index ≤ 2.2 L/min/m^2^, left ventricular ejection fraction (LVEF) ≤ 40%, or VTI LVOT ≤ 18 cm, or need for catecholamines to maintain adequate cardiac index, LVEF, or VTI LVOT;	4. Patients already on MCS before inclusion;
b. Signs of impaired organ perfusion with at least one of the following criteria:	5. Cardiogenic shock cause by viral myocarditis;
**i**. Altered mental status	6. Corticosteroid therapy within 4 weeks prior to inclusion;
**ii.** Skin mottling	7. Patient receiving one of the following treatments: ketoconazole, rifampicin, phenytoin, phenobarbital, cyclosporine, clarithromycin;
**iii.** Oliguria (≤ 25 ml/h)	8. Hypersensitivity to fludrocortisone or hydrocortisone;
**iv.** Increased serum lactate (> 2 mmol/L)	9. Pregnancy or breastfeeding;
c. Pulmonary congestion or elevated left-ventricular filling pressures	10. Privation of liberty by administrative or judicial decision;
3. Written informed consent from the patient or proxy (if present) before inclusion or once possible when patient has been included in a context of emergency;	11. Refusal of study participation or to pursue the study by the patient;
4. Benefiting of coverage by the French statutory healthcare insurance system.	

*SAP* systolic blood pressure, *MAP* mean arterial pressure, *CAHP* Cardiac Arrest Hospital Prognosis, *VTI LVOT* velocity-time integral of left ventricular outflow tract, *MCS* mechanical circulatory support

1. If the patient is capable of participating in the consent process, the investigator will obtain a written consent from the patient.

2. If the patient is unable to give his consent (e.g., comatose), the investigator will obtain a written consent from his/her legally acceptable representative as per French law. As soon as the patient will be capable of participating in the consent process, he will be given full information about the study and the investigator will obtain a continuation consent from the patient.

3. If the patient is not capable of participating in the consent process, and his/her legally acceptable representative is not present at the time of selection criteria fulfillment, the patient will be included in emergency situation as per French law. The patient or, where applicable, his/her legally acceptable representative shall be informed as soon as possible and their consent shall be sought for the possible continuation of such research.

### Randomization, comparator, and blinding

Randomization will be centralized, through a secured website, and will be stratified according to center, using permutation blocks whose size will be unknown by investigators. Patients will be randomly allocated to receive hydrocortisone plus fludrocortisone, or placebos of hydrocortisone and fludrocortisone, with double blinding. The consecutive 24 h before and after the randomization will be considered as study day-0 and day-1, respectively.

The drugs will be prepared prior to the initiation of the study and will be packed by the Pharmacology Department, *CMC Ambroise Paré*, according to the randomization list. Treatment boxes will be coded and masked centrally. Corticosteroids or placebos will be sealed in sequentially numbered, identical boxes containing 30 vials of lyophilized hydrocortisone or its placebo and 30 ampoules of injectable water and a blister package with 10 tablets of fludrocortisone or its placebo. Each box will be provided with a detachable sticker for traceability.

This study is a double-blind study: participants, clinicians, and researchers (outcome assessors and statistical analysts) are blinded to the group assignment and the blindness will never be broken prior to the completion of study unless in particular conditions. Indeed, unblinding should only be requested by the treating physician in the context of a medical emergency, when knowledge of the allocated treatment is necessary for the management of the patient.

### Withdrawal or dropout criteria

Interventions in either active group or control group will be stopped if participants meet the following criteria: (1) subject is unwilling to continue the intervention for any reasons and (2) subject suffers from any suspected unexpected serious adverse reaction during the intervention period.

### Study interventions

**Fig. 1:**
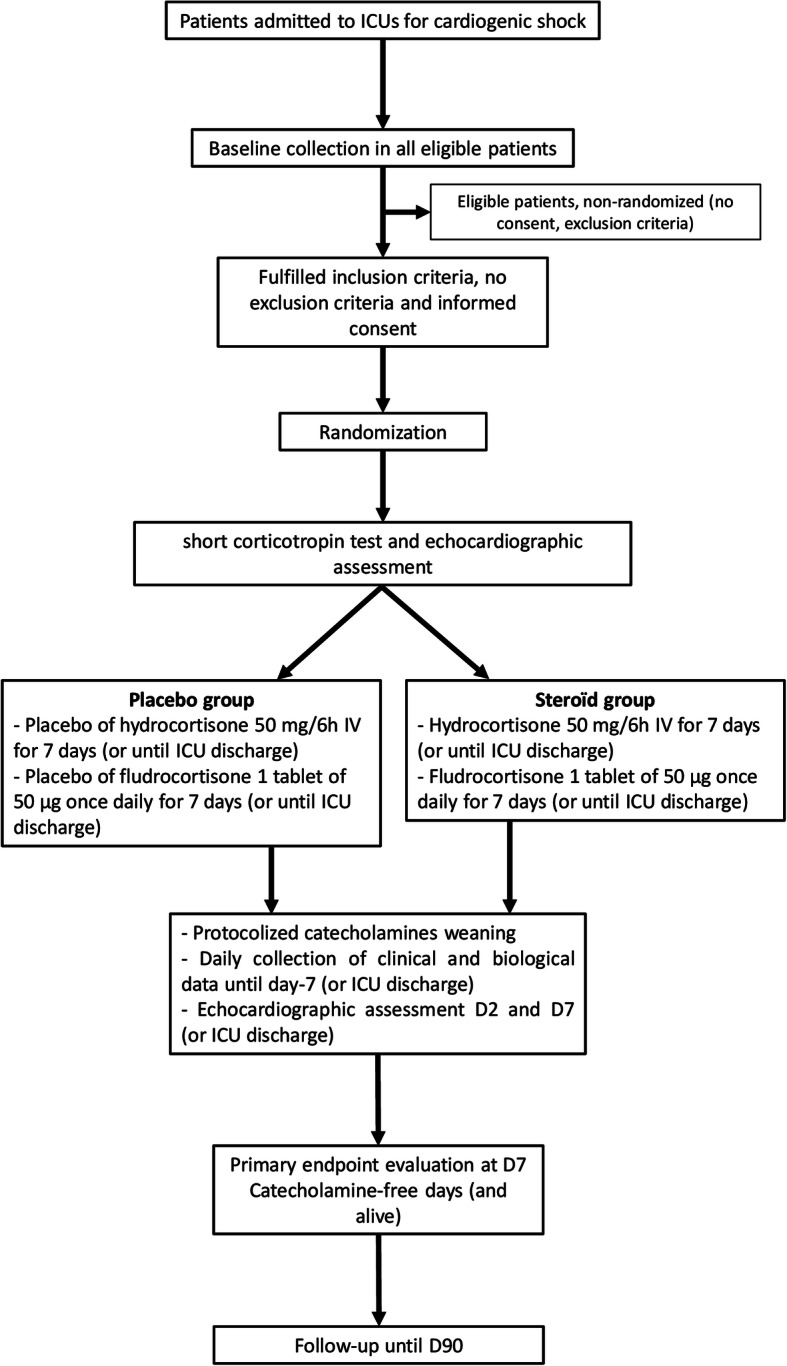
Study flow chart

The study flow chart is shown in Fig. 1. Immediately after randomization, and before study drug administration, a short corticotropin stimulation test and an echocardiographic assessment will be performed, if possible.

Patients will be randomized to intravenous infusion of hydrocortisone (50 mg/6 h) for 7 days or until discharge from the ICU, according to which event occur first) with enteral administration of fludrocortisone (tablet of 50 μg once daily in the morning for 7 days or until discharge from the ICU, according to which event occurs first) or to respective placebos. Active and placebo drugs have similar aspects.

Co-interventions will be standardized according to the French recommendations for the management of adult patients with CS [17]. The current standard is administered to both groups in addition to the intervention and placebo. Blood glucose levels will be monitored at least every 8 h and maintained at ≤ 150 mg/dl by intravenous infusion of insulin. Administration of sedative and analgesic drugs or muscle relaxants is left at the discretion of the clinician. A protocol for catecholamine weaning will be suggested to all centers in order to standardize practices. Centers where a local protocol is already in use will be authorized to continue using it. Open-labeled corticosteroids will be discouraged and no treatment will be prohibited. If there is a formal indication for corticosteroid therapy during the experimental treatment, this should be notified and justified.

### Cortisol assays

The short corticotropin stimulation test will be performed by injecting 250 mg of tetracosactrin (Synacthen®, Ciba, Reuil-Malmaison, France) intravenously as described previously [14]. The maximal post stimulation cortisol concentration (*T*_max_) is the highest value between *T*_30_ and *T*_60_. The maximal cortisol response (Δ_max_) is defined as the difference between *T*_max_ and the baseline cortisol concentration (*T*_0_). CIRCI is defined as *T*_0_ < 10 μg/dL (276 nmol/L) and/or Δmax < 9 μg/dL (248 nmol/L) [18]. We will define nonresponders to the corticotropin stimulation test as patients with Δmax < 9 μg/dL, whatever the baseline cortisol [19, 20].

## Catecholamine management

### Vasopressors

The management of vasopressors will be guided by a mean arterial pressure (MAP) target of 65–70 mmHg; this target can be individualized by the attending physician according to the patient's history (e.g., chronic arterial hypertension). In practice, as soon as MAP > 70 mmHg, it is recommended to decrease the vasopressor infusion rate in decrements of 0.05 μg/kg/min, at least every hour. If MAP > 75 mmHg, the vasopressor infusion rate is decreased in decrements of 0.1 μg/kg/min, at least every hour. In the event of hypotension or sign of impaired organ perfusion during vasopressor weaning, a return to the previous level and hemodynamic exploration will be suggested [21].

### Inotropes

The management of inotropes will be guided by cardiac index and correction of signs of hypoperfusion (e.g., mottling, oliguria < 25 ml/h, altered state of consciousness, hyperlactatemia > 2 mmol/L, central venous oxygen saturation < 60%). In practice, as soon as the tissue perfusion target is reached (and in the absence of hypotension), a decrease in dobutamine dose of 2.5 μg/kg/min will be suggested every 4 to 12 h, based on clinical evaluation. In the event of hypotension or tissue hypoperfusion during weaning, a return to the previous level and hemodynamic exploration will be suggested.

## Data collection and follow-up

Patients will be followed for 90 days (Table 2). In the data collection process, each patient is assigned a code. Data will be collected by electronic case report forms (eCRF) compliant to good clinical practice (GCP) and will be recorded on a central, secure server. Data entry will be managed by assessor blinded. Besides, each eCRF will be checked to ensure the accuracy and completion of data collection throughout the study. All personal information about potential and enrolled participants will be confidential.

**Table 2:** Enrollment, intervention, and evaluations

Time points	ICU admission to enrollment	Experimental period							Early follow-up (D7 or ICU discharge)	Follow-up	Close out
	D0	D1	D2	D3	D4	D5	D6	D7	Dx	D28	D90 + 20 days
*Enrollment*											
Eligibility screen	**•**										
Informed consent	**•**										
Randomization	**•**										
*Intervention*											
Physical examination	**•**	**•**	**•**	**•**	**•**	**•**	**•**	**•**			
Biology exams	**•**	**•**	**•**	**•**	**•**	**•**	**•**	**•**			
Echocardiography	**•**		**•**						**•**		
Corticotropin stimulation test	**•**										
Treatment		**•**	**•**	**•**	**•**	**•**	**•**	**•**			
*Assessments*											
Collection of data on the occurrence of primary endpoint									**•**		
Collection of data on the occurrence of secondary endpoints									**•**	**•**	**•**
Serious adverse events		**•**	**•**	**•**	**•**	**•**	**•**	**•**	**•**	**•**	**•**

### Baseline

Baseline data will include the following: (1) demographic and anthropometric data; (2) type of consent; (3) time of hospital and ICU admissions; (4) medical history (chronic heart failure and its cause, cardiac surgery, pacemaker, arterial hypertension, dyslipidemia, smoking, chronic obstructive pulmonary disease, chronic dialysis…); (5) etiology of CS and ventricle affected; (6) severity of illness using vital signs, Simplified Acute Physiology Score (SAPS) II [22] and sequential organ failure assessment (SOFA) score [23]; (7) type and dose of vasopressors and inotropic drugs, fluid infusion; (8) time from shock onset; (9) biological data, blood gas analyses and arterial lactate levels; (10) treatments used before randomization (etomidate, insulin, anticoagulant and antiplatelet therapies, diuretics, proton pump inhibitor, beta-blockers, mineralocorticoid receptors antagonist, renin angiotensin system inhibitors, amiodarone, levothyroxine and sedatives); (11) ventilation mode; and (12) results of the corticotropin stimulation test (baseline cortisol and Δmax).

### During treatment

We will record on a daily basis, from day 1 to day 7 or ICU discharge the following variables: (1) vital signs; (2) biological data, blood gas analyses and arterial lactate levels; (3) SOFA score [23]; (4) type and dose of vasopressor and inotropic drugs, fluid infusion; (5) other treatments used (insulin, diuretics, sedatives); (6) episodes of hyperglycemia (> 150 mg/dl); (7) administration of study drugs (hydrocortisone and fludrocortisone or placebos) and reason if it stopped; and (8) type, dose, and reason for use of any open-labeled corticosteroids.

### Echocardiographic evaluations

When possible, patients included will undergo echocardiographic assessment at randomization, day 2 and day 7 (or ICU discharge) to assess the hemodynamic changes caused by low-dose steroids with the following variables: (1) left ventricular systolic function with left ventricular ejection fraction (LVEF), systolic velocity at the mitral annulus (S-mit) and left ventricular global longitudinal strain by speckle-tracking; (2) cardiac index, obtained by measuring the velocity-time integral of left ventricular outflow tract; (3) estimation of left ventricle filling pressures, using pulsed-wave Doppler early (E) and late (A) diastolic wave velocities at the mitral valve, and tissue Doppler early (e’) diastolic wave velocity at the lateral mitral valve annulus; and (4) right ventricular function as reflected by tissue Doppler peak systolic wave at the lateral tricuspid annulus (S-tric), tricuspid annular plane systolic excursion, and estimation of systolic pulmonary arterial pressure.

### Follow-up

At day 28 and day 90, the following data will be recorded: (1) recurrence of shock (with new vasopressor and/or inotropic support); (2) mechanical circulatory support (MCS) and cardiac transplantation; (3) mechanical ventilation (MV) ; (4) specific treatment for CS (coronary reperfusion by percutaneous coronary intervention, fibrinolytic therapy, or cardiac surgery, vale replacement or repair, catheter ablation of arrhythmias, cardioversion, defibrillator or pacemaker implantation and pericardial drainage); (5) nosocomial infections; (6) renal replacement therapy; (7) other complications (bleeding, stroke, delirium, ICU-acquired neuromuscular weakness, (8) hospital and ICU length of stay; and (9) hospital and ICU mortality. Adverse events and complications will be assessed systematically.

## Endpoints

Catecholamine-free days at day 7 will be the primary endpoint. Secondary endpoints will include the following: (1) mortality at ICU and hospital discharge, day 28 and day 90; (2) ICU and hospital length of stay (days) [time frame: 28 and 90 days after randomization]; (3) total duration of catecholamine infusion (days) [time frame: 28 days after randomization] [24]*;* (4) number of days alive (up to day 28 and day 90) and free of catecholamines; (5) rate (%) of patients with need of MV [time frame: 28 days after randomization]; (6) rate (%) of patients with need of MCS [time frame: 28 days after randomization]; (7) number of days alive (up to day 7, day 28, and day 90) and without cardiovascular failure (as defined by a cardiovascular SOFA score < 3) [time frame: 7 days after randomization]; (8) lactate clearance (mmol/L) [time frame: 7 days after randomization]; (9) change in mean arterial pressure (mmHg) from the baseline [time frame: 7 days after randomization]; (10) change in cardiac index (L/min/m^2^) [time frame: 7 days after randomization]; (11) rate (%) of patients with nosocomial infection [time frame: 28 days after randomization]; (12) rate (%) of patients with need of intravenous insulin therapy [time frame: 7 days after randomization]; and (13) rate (%) of other adverse effects of corticosteroid treatment (hyperglycemia, gastrointestinal bleeding, stroke, delirium, ICU-acquired neuromuscular weakness, significant hypokalemia or hypernatremia) [time frame: 28 days after randomization].

## Subgroup analysis

We plan to explore treatment effects according to the following parameters: (1) CS secondary to acute myocardial infarction, (2) or postcardiotomy, (3) etomidate use, (4) vasopressor support, and (5) adrenal profiles (CIRCI and nonresponders to corticotropin stimulation test) [14, 18, 25].

## Sample size and statistical analysis

Based on literature [14], the primary endpoint (catecholamine-free days at day 7) is estimated at 3 ± 3 days. The increase in this number of days reasonably expected in the corticosteroid group is + 1 day [11, 12, 19]. With a bilateral formulation, 190 patients will be needed in each group (total of 380 patients), with a statistical power of 90% and an α risk of 5% to conclude to the efficacy of the experimental group.

An intention-to-treat (ITT) and a per-protocol (PP) analysis will be performed. Following the ITT principle, all randomized patients receiving at least one dose of study medication will be included in the analysis with group allocation as randomized. The PP population will be composed of patients included in the study, without major deviations from the protocol, including erroneous inclusion of patients not meeting all selection criteria, modifications or non-compliance with the initial allocated treatment, and appearance of a formal indication for corticosteroid therapy during experimental period. The primary endpoint analysis will be performed on the ITT population. Additional analyses of the primary endpoint and all secondary endpoint analyses will be performed on both the ITT and PP populations in order to describe patients excluded from the PP population and assess the impact on the ITT analysis and the robustness of the results obtained.

Any missing or invalid data will be systematically checked and searched for in the medical records of the patient concerned. In addition to the analyses of complete cases without missing data for the primary endpoint, sensitivity analyses will be performed using several methods of replacing missing data, including the Last Observation Carry Forward (LOCF) method, the worst-case scenario assumption, and multiple imputation chain equation (MICE) techniques. There are no plans to replace subjects who left the study prematurely.

No intermediate analysis is planned. STATA v14.1 (Stata Corp, College Station, TX, USA) and R 3.2.4 (R Foundation for Statistical Computing, Vienna, Austria) will be used for data analysis. The primary endpoint will be catecholamine-free days at day 7. The analysis of the primary endpoint will be carried out on the ITT population. Additional sensitivity analyses will be conducted on the PP population. Descriptive analyses will be performed. Normally distributed variables will be expressed as mean and standard deviations; non-normally distributed variables will be expressed as medians and inter quartile ranges. For categorical variables, number of patients in each category and corresponding percentages will be given. The effects of treatments on mortality rates will be compared using a logistic regression. Cumulative event curves (censored endpoints) will be estimated with Kaplan–Meier procedure, and Cox model will be used to compare treatments effects. Analysis of variance will be used to compare number of days alive and free of vasopressors, MV, and with a cardiovascular SOFA score < 3. The same analyses will be conducted for subgroups unless the numbers of patients are insufficient. In this case, statistical methods will be adapted according to sample sizes.

## Adverse event reporting

During hospitalization, patients are closely monitored for adverse events (AE) and serious adverse events (SAEs). Expected AEs are those that are anticipated in the population under study, regardless of participation in research. Expected SAEs in this study include the following: (1) severe infection (life-threatening, leading to death or septic shock); (2) refractory shock (defined as the need for MCS); (3) vascular disorders: stroke, myocardial infarction, peripheral arterial ischemia; (4) hypokalemia (< 3 mmol/L) and its consequences, if the event occurs between day 1 and day 8; (5) hypersensitivity reaction (including anaphylactic reaction); and (6) death from any cause. Any adverse event that meets a protocol-defined criterion as a SAE will be reported electronically to the data coordinating center without delay (within 24 h of site awareness) on SAE form. Moreover, suspected unexpected serious adverse reactions (SUSARs) will be registered. At any time after completion of the study, if the principal investigator or appropriate co-investigator becomes aware of an SAE, the principal investigator or appropriate co-investigator will report the event to the designated Pharmacovigilance Group. The intensity of adverse events will assess by the investigator according to the CTC-AE V4.0 classification. Safety analyses, as part of the endpoints, will be based on the safety set, consisting of the pre-defined SAEs, and will comprise standard descriptive methods.

## Study organization

The financial support and study promotion will be performed by *CMC Ambroise Paré*. An independent Data and Safety Monitoring Board (DSMB), that is not involved in the design and conduct of the trial, and has no affiliation with the sponsor, looks over the ethics in accordance with the Declaration of Helsinki, monitors patient safety, and reviews safety issues as the study progresses. SAEs and unexpected related or possibly related serious events are reported blindly to the DSMB. The sponsor has a liability insurance which is in accordance with national legislation, guaranteeing its own liability as well as that of the investigators. In accordance with applicable regulations and GCP, a study monitor is periodically controlling study procedures. A trial steering committee (TSC) will monitor the overall conduct of the trial. The TSC will make recommendations regarding all trial-related decisions including those based on recommendations from the DSMB.

## Protocol amendments

Important protocol modification (i.e., inclusion and exclusion criteria, outcome, analyses) will be informed to the Independent Ethics committee (*Comité de Protection des Personnes CPP— Ile-de-France IV*).

## Dissemination of research results

After completion of the study, its findings will be published in some international peer-reviewed journals and presented at national/international scientific conferences. Both positive and negative results will be published. A summary of the results will be made available to the study patients if requested. Concerning authorship, all researchers and other colleagues who participated in this study will be co-authors or collaborators of the study based on their individual contributions. All authors who fulfill the authorship criteria will be included in future publications. There is no intended use of professional medical writers. The full protocol, participant-level dataset, and statistical code will be available from the corresponding author on reasonable request.

## Discussion

The COCCA study is the first randomized controlled trial to investigate low dose of corticosteroid therapy in adult patients with CS. This trial investigates the hemodynamic effects of hydrocortisone and fludrocortisone and replicates as much as possible the intervention of APROCCHSS trial in septic shock patients [12].

Corticosteroids have been considered in the management of several acute illnesses [18, 26]. During septic shock, it is now clear that the use of low-dose corticosteroids enhance shock reversal [7–9], with acceptable side effects as hyperglycemia and hypernatremia, and potential survival benefits [9]. These results were recently confirmed by APROCCHSS and ADRENAL trials [11, 12]. Corticosteroids have cardiovascular effects during shock, with an increase in mean arterial pressure, and systemic vascular resistance [27, 28]. Corticosteroids can improve vasopressor sensibility [20], especially in case of adrenal dysfunction [29, 30], through an increase in α adrenergic receptor gene expression [31] and endothelial glucocorticoid receptors [32]. Additionally, corticosteroids probably improve cardiac function [33–38], especially in patients with circulatory shock with long-term catecholamine treatment [39, 40]. Given the many similarities between CS and septic shock with a SIRS-like pathophysiology, we can expect a hemodynamic effect of low-dose corticosteroids in CS similar to that in septic shock. Moreover, it seems interesting to identify subgroups of patients more likely to benefit from corticosteroids [19] and those at high risk of harm [41]. This study will attempt to provide robust answers about the usefulness of corticosteroid replacement therapy in adult with CS [42].

## Risk of bias and study limitations

The risks of bias of the COCCA study were minimized by a robust randomization (computer-generated and stratified by center) and a strong double-blind (with double placebo) design. We have chosen as comparator placebo in order to control the placebo effect and to minimize the potential bias resulting from differences in management, especially catecholamine weaning because of the nature of the primary endpoint (catecholamine-free days at day-7). Concerning our experimental arm, we have selected a corticosteroid regimen consisting of fludrocortisone plus hydrocortisone, because the only two RCTs showing a benefit on mortality used this combination in septic shock [12, 19]. Moreover, hydrocortisone was administrated as intermittent intravenous bolus and without taper off in these studies, as in ours. Meta-analyses have demonstrated that the administration method of hydrocortisone (intermittent bolus vs. continuous infusion) did not seem to influence the mortality, in contrast to the use of taper off [9].

The COCCA trial potentially includes some limitations. Firstly, the primary endpoint, catecholamine-free days at day 7, was chosen in part because an all-cause mortality endpoint would not have been realistic. Indeed, APROCCHSS and ADRENAL studies initially planned to analyze 1280 and 3800 patients, respectively, to detect a small difference in mortality. Second, we will exclude patients already undergoing MCS at time of inclusion, in whom the potential benefit of substitutive corticosteroid therapy is possibly high. Thirdly, exposure to etomidate in some patients could be problematic. Indeed, it is an adrenal suppressant drug, but it is also a widely used hypnotic agent for urgent intubation in CS. We have planned a subgroup analysis taking into account etomidate use. Last, the spectrum of patients with CS included is wide. Therefore, the most specific phenotypes of CS (acute myocardial infarction, postcardiotomy, need of vasopressor support) will be assessed by subgroup analyses.

In conclusion, the COCCA trial should help assess whether low dose corticosteroids are beneficial in adult patients with CS. This pragmatic study is the first large RCT focusing on substitutive corticosteroid therapy in CS.

## Trial status

This is version 9.0 of the protocol, dated 22 February 2021. The first patient was randomized on April 19, 2019. Recruitment is predicted to continue until April 2022. At the time of submission, participant recruitment is still ongoing.

## Data Availability

Dataset of this study will be available through the corresponding author if there is a reasonable request.

## Abbreviations

CIRCI: Critical illness-related corticosteroid insufficiency
HPA: Hypothalamo-pituitary-adrenal
ACTH: Adrenocorticotropic hormone
COCCA trial: COrticosteroid in Cardiogenic shoCk in Adult patient trial
ICU: Intensive care unit
*T*_max_: Maximal post stimulation cortisolemia concentration
Δ_max_: Maximal cortisol response
*T*_0_: Baseline cortisolemia
MAP: Mean arterial pressure
eCRF: Electronic case report forms
GCP: Compliant to good clinical practice
SAPS: Simplified Acute Physiology Score
SOFA score: Sequential organ failure score
RAS: Renin angiotensin system
LVEF: Left ventricular ejection fraction
S-mit: Systolic velocity at the mitral annulus
LV-GLS: Left ventricular global longitudinal strain
VTI: Velocity-time integral
LVOT: Left ventricular outflow tract
E: Early diastolic wave velocities at the mitral valve
A: Late diastolic wave velocities at the mitral valve
e’: Tissue Doppler early diastolic wave velocity at the lateral mitral valve annulus
S-tric: Systolic right ventricular function by tissue Doppler peak systolic wave at the lateral tricuspid annulus
TAPSE: Tricuspid annular plane systolic excursion
sPAP: Systolic pulmonary arterial pressure
MCS: Mechanical circulatory support
MV: Mechanical ventilation
ITT: Intention-to-treat
PP: Per-protocol
AE: Adverse events
SAE: Serious adverse events
SUSAR: Suspected unexpected serious adverse reactions
DSMB: Data and safety monitoring board
TSC: Trial steering committee
RCT: Randomized controlled trial

## References

[1] Puymirat E, Fagon JY, Aegerter P, Diehl JL, Monnier A, Hauw-Berlemont C, Boissier F, Chatellier G, Guidet B, Danchin N, Aissaoui N, on behalf of the Collège des Utilisateurs de Bases de données en Réanimation (CUB‐Réa Group [Intensive Care Database User Group]) (2017). Cardiogenic shock in intensive care units: evolution of prevalence, patient profile, management and outcomes, 1997-2012. Eur J Heart Fail..

[2] van Diepen S, Katz JN, Albert NM, Henry TD, Jacobs AK, Kapur NK, Kilic A, Menon V, Ohman EM, Sweitzer NK, Thiele H, Washam JB, Cohen MG, American Heart Association Council on Clinical Cardiology; Council on Cardiovascular and Stroke Nursing; Council on Quality of Care and Outcomes Research; and Mission: Lifeline (2017). Contemporary management of cardiogenic shock: a scientific statement from the American Heart Association. Circulation..

[3] Hochman JS, Sleeper LA, Webb JG, Sanborn TA, White HD, Talley JD, Buller CE, Jacobs AK, Slater JN, Col J, McKinlay SM, Picard MH, Menegus MA, Boland J, Dzavik V, Thompson CR, Wong SC, Steingart R, Forman R, Aylward PE, Godfrey E, Desvigne-Nickens P, LeJemtel TH (1999). Early revascularization in acute myocardial infarction complicated by cardiogenic shock. SHOCK Investigators. Should we emergently revascularize occluded coronaries for cardiogenic shock. N Engl J Med..

[4] Hochman JS (2003). Cardiogenic shock complicating acute myocardial infarction: expanding the paradigm. Circulation..

[5] Marik PE, Pastores SM, Annane D, Meduri GU, Sprung CL, Arlt W, Keh D, Briegel J, Beishuizen A, Dimopoulou I, Tsagarakis S, Singer M, Chrousos GP, Zaloga G, Bokhari F, Vogeser M, American College of Critical Care Medicine (2008). Recommendations for the diagnosis and management of corticosteroid insufficiency in critically ill adult patients: consensus statements from an international task force by the American College of Critical Care Medicine. Crit Care Med..

[6] Annane D, Pastores SM, Arlt W, Balk RA, Beishuizen A, Briegel J, Carcillo J, Christ-Crain M, Cooper MS, Marik PE, Meduri GU, Olsen KM, Rochwerg B, Rodgers SC, Russell JA, van den Berghe G (2017). Critical illness-related corticosteroid insufficiency (CIRCI): a narrative review from a Multispecialty Task Force of the Society of Critical Care Medicine (SCCM) and the European Society of Intensive Care Medicine (ESICM). Intensive Care Med..

[7] Rygård SL, Butler E, Granholm A, Møller MH, Cohen J, Finfer S, Perner A, Myburgh J, Venkatesh B, Delaney A (2018). Low-dose corticosteroids for adult patients with septic shock: a systematic review with meta-analysis and trial sequential analysis. Intensive Care Med..

[8] Fang F, Zhang Y, Tang J, Lunsford LD, Li T, Tang R, He J, Xu P, Faramand A, Xu J, You C (2019). Association of corticosteroid treatment with outcomes in adult patients with sepsis: a systematic review and meta-analysis. JAMA Intern Med..

[9] Annane D, Bellissant E, Bollaert PE, Briegel J, Keh D, Kupfer Y, Pirracchio R, Rochwerg B, Cochrane Emergency and Critical Care Group (2019). Corticosteroids for treating sepsis in children and adults. Cochrane Database Syst Rev..

[10] Rochwerg B, Oczkowski SJ, Siemieniuk RAC, Agoritsas T, Belley-Cote E, D’Aragon F, Duan E, English S, Gossack-Keenan K, Alghuroba M, Szczeklik W, Menon K, Alhazzani W, Sevransky J, Vandvik PO, Annane D, Guyatt G (2018). Corticosteroids in sepsis: an updated systematic review and meta-analysis. Crit Care Med..

[11] Venkatesh B, Finfer S, Cohen J, Rajbhandari D, Arabi Y, Bellomo R, Billot L, Correa M, Glass P, Harward M, Joyce C, Li Q, McArthur C, Perner A, Rhodes A, Thompson K, Webb S, Myburgh J, ADRENAL Trial Investigators and the Australian–New Zealand Intensive Care Society Clinical Trials Group (2018). Adjunctive glucocorticoid therapy in patients with septic shock. N Engl J Med..

[12] Annane D, Renault A, Brun-Buisson C, Megarbane B, Quenot J-P, Siami S, Cariou A, Forceville X, Schwebel C, Martin C, Timsit JF, Misset B, Ali Benali M, Colin G, Souweine B, Asehnoune K, Mercier E, Chimot L, Charpentier C, François B, Boulain T, Petitpas F, Constantin JM, Dhonneur G, Baudin F, Combes A, Bohé J, Loriferne JF, Amathieu R, Cook F, Slama M, Leroy O, Capellier G, Dargent A, Hissem T, Maxime V, Bellissant E, CRICS-TRIGGERSEP Network (2018). Hydrocortisone plus fludrocortisone for adults with septic shock. N Engl J Med..

[13] Bagate F, Razazi K, Boissier F, Seemann A, de Prost N, Carteaux G, Brun-Buisson C, Mekontso Dessap A (2017). Association between relative adrenal insufficiency and septic cardiomyopathy: a preliminary report. Intensive Care Med..

[14] Bagate F, Lellouche N, Lim P, Moutereau S, Razazi K, Carteaux G, de Prost N, Dubois-Randé JL, Brun-Buisson C, Mekontso Dessap A (2017). Prognostic value of relative adrenal insufficiency during cardiogenic shock: a prospective cohort study with long-term follow-up. Shock..

[15] Ducrocq N, Biferi P, Girerd N, Latar I, Lemoine S, Perez P, Thivilier C, Levy B, Kimmoun A (2018). Critical illness-related corticosteroid insufficiency in cardiogenic shock patients: prevalence and prognostic role. Shock Augusta Ga..

[16] Maupain C, Bougouin W, Lamhaut L, Deye N, Diehl J-L, Geri G, Perier MC, Beganton F, Marijon E, Jouven X, Cariou A, Dumas F (2016). The CAHP (Cardiac Arrest Hospital Prognosis) score: a tool for risk stratification after out-of-hospital cardiac arrest. Eur Heart J..

[17] Levy B, Bastien O, Karim B, Cariou A, Chouihed T, Combes A (2015). Experts’ recommendations for the management of adult patients with cardiogenic shock. Ann Intensive Care..

[18] Annane D, Pastores SM, Rochwerg B, Arlt W, Balk RA, Beishuizen A, Briegel J, Carcillo J, Christ-Crain M, Cooper MS, Marik PE, Umberto Meduri G, Olsen KM, Rodgers S, Russell JA, van den Berghe G (2017). Guidelines for the diagnosis and management of critical illness-related corticosteroid insufficiency (CIRCI) in critically ill patients (Part I): Society of Critical Care Medicine (SCCM) and European Society of Intensive Care Medicine (ESICM) 2017. Intensive Care Med..

[19] Annane D, Sebille V, Charpentier C, Bollaert PE, Francois B, Korach JM (2002). Effect of treatment with low doses of hydrocortisone and fludrocortisone on mortality in patients with septic shock. JAMA..

[20] Annane D, Bellissant E, Sebille V, Lesieur O, Mathieu B, Raphael JC, Gajdos P (1998). Impaired pressor sensitivity to noradrenaline in septic shock patients with and without impaired adrenal function reserve. Br J Clin Pharmacol..

[21] Asfar P, Meziani F, Hamel JF, Grelon F, Megarbane B, Anguel N, Mira JP, Dequin PF, Gergaud S, Weiss N, Legay F, le Tulzo Y, Conrad M, Robert R, Gonzalez F, Guitton C, Tamion F, Tonnelier JM, Guezennec P, van der Linden T, Vieillard-Baron A, Mariotte E, Pradel G, Lesieur O, Ricard JD, Hervé F, du Cheyron D, Guerin C, Mercat A, Teboul JL, Radermacher P, SEPSISPAM Investigators (2014). High versus low blood-pressure target in patients with septic shock. N Engl J Med..

[22] Le Gall JR, Lemeshow S, Saulnier F (1993). A new Simplified Acute Physiology Score (SAPS II) based on a European/North American multicenter study. JAMA..

[23] Vincent JL, de Mendonca A, Cantraine F, Moreno R, Takala J, Suter PM, Sprung CL, Colardyn F, Blecher S (1998). Use of the SOFA score to assess the incidence of organ dysfunction/failure in intensive care units: results of a multicenter, prospective study. Working group on “sepsis-related problems” of the European Society of Intensive Care Medicine. Crit Care Med..

[24] Gaies MG, Gurney JG, Yen AH, Napoli ML, Gajarski RJ, Ohye RG, Charpie JR, Hirsch JC (2010). Vasoactive-inotropic score as a predictor of morbidity and mortality in infants after cardiopulmonary bypass. Pediatr Crit Care Med..

[25] Annane D, Sebille V, Troche G, Raphael JC, Gajdos P, Bellissant E (2000). A 3-level prognostic classification in septic shock based on cortisol levels and cortisol response to corticotropin. JAMA..

[26] Pastores SM, Annane D, Rochwerg B, Corticosteroid Guideline Task Force of S, Esicm (2017). Guidelines for the diagnosis and management of critical illness-related corticosteroid insufficiency (CIRCI) in critically ill patients (Part II): Society of Critical Care Medicine (SCCM) and European Society of Intensive Care Medicine (ESICM) 2017. Intensive Care Med..

[27] Bellissant E, Annane D (2000). Effect of hydrocortisone on phenylephrine--mean arterial pressure dose-response relationship in septic shock. Clin Pharmacol Ther..

[28] Keh D, Boehnke T, Weber-Cartens S, Schulz C, Ahlers O, Bercker S, Volk HD, Doecke WD, Falke KJ, Gerlach H (2003). Immunologic and hemodynamic effects of “low-dose” hydrocortisone in septic shock: a double-blind, randomized, placebo-controlled, crossover study. Am J Respir Crit Care Med..

[29] Aboab J, Polito A, Orlikowski D, Sharshar T, Castel M, Annane D (2008). Hydrocortisone effects on cardiovascular variability in septic shock: a spectral analysis approach. Crit Care Med..

[30] Grunfeld JP, Eloy L (1987). Glucocorticoids modulate vascular reactivity in the rat. Hypertension..

[31] Sakaue M, Hoffman BB (1991). Glucocorticoids induce transcription and expression of the alpha 1B adrenergic receptor gene in DTT1 MF-2 smooth muscle cells. J Clin Invest..

[32] Goodwin JE, Feng Y, Velazquez H, Sessa WC (2013). Endothelial glucocorticoid receptor is required for protection against sepsis. Proc Natl Acad Sci U S A..

[33] Lefer AM (1967). Factors influencing the inotropic effect of corticosteroids. Proc Soc Exp Biol Med Soc Exp Biol Med N Y N..

[34] Nishimura H, Yoshikawa T, Kobayashi N, Anzai T, Nagami K, Handa S, Ogawa S (1997). Effects of methylprednisolone on hemodynamics and beta-adrenergic receptor signaling in rabbits with acute left ventricular failure. Heart Vessels..

[35] Ouvrard-Pascaud A, Sainte-Marie Y, Bénitah J-P, Perrier R, Soukaseum C (2005). Nguyen Dinh Cat A, et al. Conditional mineralocorticoid receptor expression in the heart leads to life-threatening arrhythmias. Circulation..

[36] Oakley RH, Ren R, Cruz-Topete D, Bird GS, Myers PH, Boyle MC, Schneider MD, Willis MS, Cidlowski JA (2013). Essential role of stress hormone signaling in cardiomyocytes for the prevention of heart disease. Proc Natl Acad Sci U S A..

[37] Oakley RH, Cidlowski JA (2015). Glucocorticoid signaling in the heart: a cardiomyocyte perspective. J Steroid Biochem Mol Biol..

[38] Cruz-Topete D, Myers PH, Foley JF, Willis MS, Cidlowski JA (2016). Corticosteroids are essential for maintaining cardiovascular function in male mice. Endocrinology..

[39] Saito T, Takanashi M, Gallagher E, Fuse A, Suzaki S, Inagaki O, Yamada K, Ogawa R (1995). Corticosteroid effect on early beta-adrenergic down-regulation during circulatory shock: hemodynamic study and beta-adrenergic receptor assay. Intensive Care Med..

[40] Saito T, Fuse A, Gallagher ET, Cutler S, Takanashi M, Yamada K, Carlsson C, Carney E, Abou-Say FK, Ogawa R (1996). The effect of methylprednisolone on myocardial beta-adrenergic receptors and cardiovascular function in shock patients. Shock..

[41] Antcliffe DB, Burnham KL, Al-Beidh F, Santhakumaran S, Brett SJ, Hinds CJ (2019). Transcriptomic signatures in sepsis and a differential response to steroids. From the VANISH Randomized Trial. Am J Respir Crit Care Med..

[42] Briegel J, Mohnle P, Vogeser M, Thiemermann C, Radermacher P (2017). Relative adrenal insufficiency in cardiogenic shock: is there a need for action?. Shock..

